# Spinal gout causing reversible quadriparesis: a case report and literature review

**DOI:** 10.1080/20009666.2018.1472515

**Published:** 2018-06-12

**Authors:** Jaspreet Kaler, Osama Mukhtar, Mazin Khalid, Shivani Thapa, Ravinder Kaler, Brandon Ting, Vijay Gayam

**Affiliations:** a Department of Medicine, Interfaith Medical Center, Brooklyn, USA; b Caribbean Medical University, Curacao, Curacao; c Avalon University School of Medicine, Curacao, Curacao

**Keywords:** Spinal gout, tophi, quadriparesis, back pain, uric acid

## Abstract

Gout commonly affects peripheral joints and is rarely found in axial joints, such as the spine and sacroiliac joints. We report a case of a patient that presented with quadriparesis who was empirically treated for spinal gout and a review of relevant literature. A 77-year-old male presented with new-onset quadriparesis that developed over 3 days. MRI imaging was suggestive of tophaceous gout of the cervical spine, but our patient refused a spinal biopsy. He was empirically treated with high-dose steroids and his upper and lower extremities weakness started improving within 3 days and resolved completely. Although spinal gout is uncommon, this case indirectly suggests that gout should be kept as a differential diagnosis when faced with back pain or quadriparesis. This case implies that empiric treatment should be considered when radiographic evidence is suggestive of tophaceous gout of the spine.

## 1. Introduction

Gout is caused by deposition of monosodium urate crystals in joints, bones, and soft tissue. It can present as recurrent inflammatory arthritis, chronic arthropathy, as urate deposits called tophi, nephrolithiasis, and chronic nephropathy. It presents earlier in males than in females and is rarely seen in children. It has an estimated prevalence of over eight million people in the US [1]. Typically, gout presents with pain, erythema, warmth and swelling in the peripheral joints, and is uncommon in axial joints such as the spine and sacroiliac joints. We present a case of reversible quadriparesis in a patient with a longstanding history of gout who was empirically treated for spinal gout based on radiographic evidence suggestive of tophi in the cervical spine.

## 2. Case report

A 77-year-old, African-American male, with past medical history of hypertension, untreated hepatitis C infection, and an over 20-year history of gout, came to the ED with complaints of progressive upper and lower extremity weakness for a duration of 3 days. He reported he was in his usual state of health 3 days ago and was able to ambulate without difficulty. Over the past 3 days, his weakness progressed to the point where he was unable to get out of bed. He denied any history of trauma, fall, fever, chills, or night sweats. His examination was significant for muscle strength of 3/5 in bilateral lower extremities, while he had strength of 4/5 in his upper extremities. He had no other neurological findings, including intact sensation and normal rectal sphincter tone. No tophi were seen on examination, but he did have minimal tenderness upon palpation of bilateral wrists and bilateral first metatarsal joints.

Laboratory workup revealed leukocytosis of 16.7 × 10^3^µL (reference range 4.5−11.0 × 10^3^) with 85% neutrophils, urate level of 6.6mg/dL (reference range 3.7–8.6), CRP of 266.2mg/L (reference range 0–4.0), ESR of 109mm/hr (reference range 0–20), BUN of 18mg/dL (reference range 8–20), and creatinine of 1.4mg/dL (reference range 0.4–1.3).

**Figure 1.**
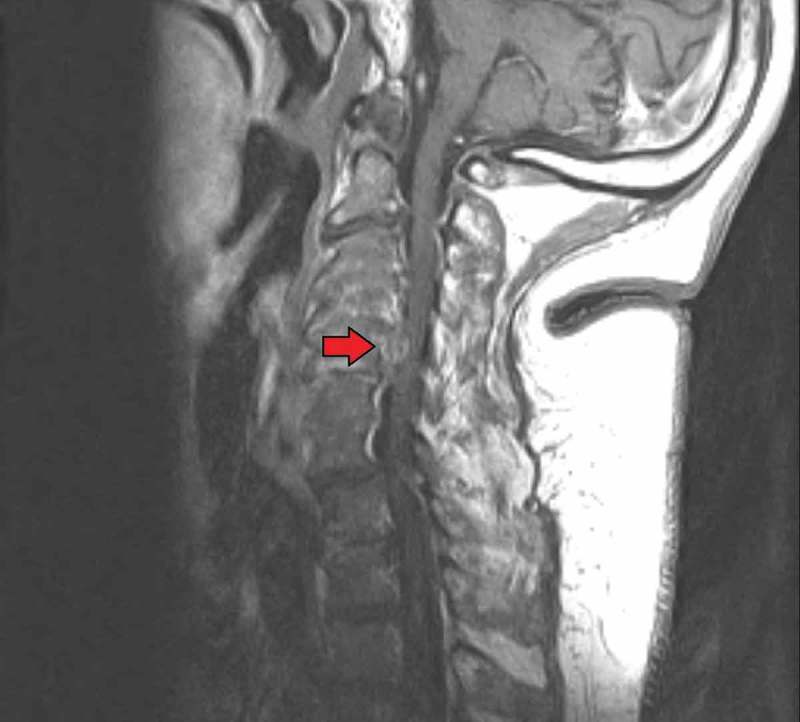
Sagittal section of cervical spine magnetic resonance image (MRI) showing mass on the inferior border of the C5 epidural space.

**Figure 2.**
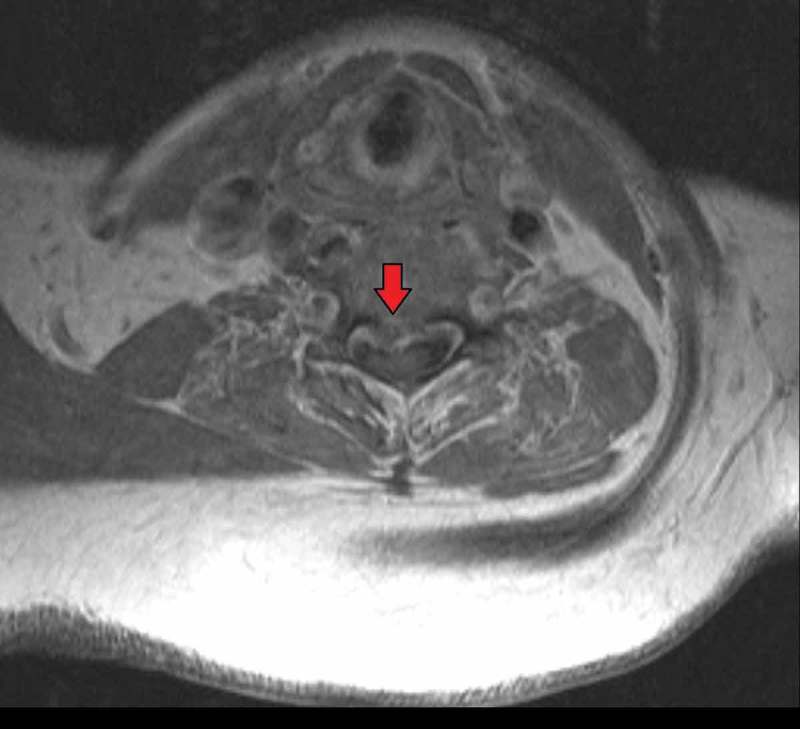
Axial section of cervical spine showing a mass with enhancement and causing cord compression.

As he had presented with tachycardia, low-grade fever of 100.4°F, and leukocytosis, we attempted to find an infectious source to explain his history and presentation. Chest X-ray and urinalysis were obtained and ruled out pneumonia or urinary tract infection. Two blood culture sets were sent on admission and were ultimately reported as negative. He had no abdominal complaints to signify any abdominal pathology. CT of the spine without IV contrast was done to look for a spinal source. No contrast was given as he had acute kidney injury on admission. The CT revealed degenerative joint disease, but no abscess or mass was seen. Ultimately, cervical spine MRI with gadolinium was done. The MRI revealed a homogenous density on the inferior border of the C5 epidural space with low signal intensity on T1 and T2 images causing compression of the spinal cord (Figures 1 and 2). This density was separate from the vertebra-disc complex and unlikely to be an osteophyte when discussed with the in-house radiologist. These changes were significant for tophi in the cervical spine causing quadriparesis in our patient.

The patient was offered a biopsy to confirm the suspicion of spinal gout and possible neurosurgical intervention. However, the patient refused any such procedure. He was then empirically started on high-dose steroids for 3 days and then tapered down and switched to oral prednisone. His upper extremities weakness resolved and lower extremities strength improved to 4/5 by day 3 of intravenous steroids. He was then discharged on oral prednisone and sent to an acute rehabilitation facility. Our patient was later seen in our associated medical clinic 1 month later and at that time his upper and lower extremities muscle strength was 5/5, and he was ambulatory. Uric acid level done at that time was 10.5mg/dL and he was started on Allopurinol.

## 3. Discussion

The pathophysiology for why certain individuals develop gout is multifactorial. It is based on genetics, various causes of hyperuricemia and innate immune processes. Hyperuricemia can occur due to decreased renal uric acid excretion or increased uric acid production. Non-modifiable risk factors that increase the risk for hyperuricemia include male gender, postmenopausal status, advanced age, or Pacific Islander descent. Modifiable risk factors for hyperuricemia include obesity, a diet with high meat or seafood content, alcohol, drinks with high fructose or sucrose, hypertension, chronic kidney disease, organ transplant recipients, and certain medications such as thiazide or loop diuretics. Although these risk factors increase hyperuricemia, not all individuals will develop signs or symptoms of gout, partially due to genetics. In fact, 28 specific genome loci have already been identified to be associated with gout (TRIM46, INHBB, SFMBT1, TMEM171, VEGFA, BAZ1B, PRKAG2, STC1, HNF4G, A1CF, ATXN2, UBE2Q2, IGF1R, NFAT5, MAF, HLF, ACVR1B-ACVRL1, and B3GNT4) [2].

Tophi seen in spinal gout are a consequence of monosodium urate crystals encompassed by inflammation. The specific locations of tophi formation occur as a consequence of pH, temperature, trauma, and a nucleating agent that can be found in synovial fluid [3–5]. The exact specific cause of tophi in spinal gout is unclear. It has been suggested that poor vascularization of spinal disc cells in patients with risk factors such as dyslipidemia, obesity, and a sedentary lifestyle, creates an environment for tophi deposition [6]. Furthermore, if a patient has degenerative joint disease of the spine it decreases oxygen and creates an acidic area with increased risk for deposition of monosodium urate crystals [6]. In fact, our patient had dyslipidemia and degenerative joint disease, both making him a candidate at risk for spinal tophi.

Review of literature has shown that the earliest case of tophaceous spinal gout was reported as early as 1950, although this was discovered postmortem in a patient with known history of gout and neck stiffness [7]. By 1976, three more cases of spinal gout were reported that required neurosurgical intervention of the ligamentum flavum due to urate deposition causing neurological compromise [8]. By 2015, the number of reported cases of spinal gout has increased to 131, with more than 50 percent involving the lumbar spine [9]. The reported cases revealed that many spinal regions can be affected by gout [10]. This includes vertebral bodies, pedicules, laminae, interapophyseal cartilage, epidural spaces and intradural spaces [10]. In the comprehensive review of all spinal gout cases done by Toprover *et al* in 2015, 131 cases were reviewed including a comparison of diagnosis and treatment courses [9]. Eleven of the 131 cases were diagnosed clinically without biopsy confirmation, similar to our patient [9]. Of the total 131 reported cases, 37% were treated with only medical management, with 45% of those cases having complete symptom resolution, similar to our patient [9].

Symptoms of axial gout vary from fever, localized pain, spinal nerve root compression, spinal cord compression, cranial nerve palsy and even atlanto-axial subluxation [10]. Diagnosis of tophaceous spinal gout is extremely difficult in that patients usually present with nonspecific symptoms and the most accurate test to confirm its diagnosis is a needle biopsy with histology. Plain X-ray findings are nonspecific. CT findings suggestive of gout include lobular juxta-articular masses with a density greater than the surrounding area or well-defined intra- and juxta-articular erosions with sclerotic borders [11]. MRI findings suggestive of gout include homogenous low-to-intermediate signal intensity on T1 and variable intensity on T2-weighted imaging, with enhancement with gadolinium [11]. Our patient had low signal intensity on T1 and T2-weighted imaging, compatible with spinal gout. Dual-energy CT is another imaging modality that can be used in patients who refuse biopsy, such as this patient, but it was unavailable at our facility. It allows the visualization of tophi as its chemical properties differ from others. It has a sensitivity of 91.9% and specificity of 85.4%, but it is costly and not widely available [12].

Treatment of gout is based on decreasing inflammation, decreasing uric acid and trying to dissolve tophi causing symptoms, especially if spinal tophi are present. In acute flares, non-steroidal anti-inflammatory drugs, colchicine or glucocorticoids can be used. For long-term management of hyperuricemia, urate-lowering agents such as allopurinol, febuxostat, probenecid and lesinurad should be used. Arhalofenate is a novel uricosuric that has shown efficacy in lowering uric acid in phase 2 trials and is not currently available [13]. Pegloticase and rasburicase are also available to lower uric acid by providing urate oxidase to convert uric acid to a more soluble product [14]. This allows for decrease in the size of tophi when other urate lowering agents have been ineffective or are contraindicated [14].

## 4. Conclusion

Traditionally, gout is common and commonly affects peripheral joints causing pain, warmth, erythema, and swelling. Gout in axial joints such as the spine and sacroiliac joints is rarely reported. This may be due to the fact that complaints of back pain and neuropathy are common, yet nonspecific. Spinal gout is difficult to diagnose in common imaging that is performed for these complaints. Although uncommon, spinal gout should be kept as a differential diagnosis when faced with back pain or quadriparesis in the correct clinical setting. This case suggests that empiric treatment should be considered when radiographic evidence is suggestive of tophaceous gout of the spine.
